# Simple and Fast Method for Fabrication of Endoscopic Implantable Sensor Arrays

**DOI:** 10.3390/s140711416

**Published:** 2014-06-26

**Authors:** I. Bogachan Tahirbegi, Margarita Alvira, Mònica Mir, Josep Samitier

**Affiliations:** 1 Nanobioengineering Laboratory, Institute for Bioengineering of Catalonia (IBEC), Baldiri Reixac, 10-12, Barcelona 08028, Spain; E-Mails: malvira@ibecbarcelona.eu (M.A.); mmir@ibecbarcelona.eu (M.M.); 2 The Biomedical Research Networking center in Bioengineering, Biomaterials and Nanomedicine (CIBER-BBN), Maria de Luna, 11, 50018, Zaragoza, Spain; E-Mail: jsamitier@ibecbarcelona.eu; 3 Department of Electronics, University of Barcelona (UB), Martí I Franques, 1, Barcelona 08028, Spain

**Keywords:** ion-selective electrode (ISE) sensor, pH-nitrate detection, electrochemistry, implantable device, biomedicine

## Abstract

Here we have developed a simple method for the fabrication of disposable implantable all-solid-state ion-selective electrodes (ISE) in an array format without using complex fabrication equipment or clean room facilities. The electrodes were designed in a needle shape instead of planar electrodes for a full contact with the tissue. The needle-shape platform comprises 12 metallic pins which were functionalized with conductive inks and ISE membranes. The modified microelectrodes were characterized with cyclic voltammetry, scanning electron microscope (SEM), and optical interferometry. The surface area and roughness factor of each microelectrode were determined and reproducible values were obtained for all the microelectrodes on the array. In this work, the microelectrodes were modified with membranes for the detection of pH and nitrate ions to prove the reliability of the fabricated sensor array platform adapted to an endoscope.

## 1. Introduction

The development of miniaturized, implantable chemical sensors to monitor different diseases in organs or blood-gas measuring pH, glucose, lactate and electrolytes such as Na^+^, K^+^ and Ca^2+^ remain one of the great challenges in analytical science. Currently, there is progress in the development of implantable chemical sensors capable of real-time monitoring of clinically important analytes such as pH, glucose and lactate in blood [1]. Ion-selective electrodes (ISEs) are a very promising approach to fabricate implantable chemical sensors to detect analytes for their low cost and easy miniaturization. They have been used in a wide variety of applications, ranging from environmental analysis [2,3] to biomedical procedures [4,5]. In a classical ISE arrangement, an ion-selective membrane is used to separate the sample solution from an internal solution, into which the working and reference electrodes are dipped. However, for applications in which miniaturized sensors are needed such as in the case of implantable sensors/devices, elimination of the internal solution is required. The best approach to achieve a solution-free arrangement (the so called all-solid-state sensors) is to adhere an ion selective membrane, usually based on polyvinyl chloride (PVC), directly on the working electrode.

The manufacturing of miniature ISE (and in general microelectrodes) arrays involves relatively complex techniques, such as screen printing, injection molding or photolithography, which require clean room facilities. Photolithography is a microfabrication process that consists of patterning parts of a thin film or the bulk of a substrate. UV light is used to transfer a geometric pattern from a photomask to a photoresist (light-sensitive chemical) deposited on the substrate. A series of chemical treatments then allows removal of the photoresist from the exposed (positive photolithography) or the unexposed (negative photolithography) areas and permits deposition of a new material in the desired pattern. The biggest advantage of this technique is that the size, shape, and spacing of an electrode array may be down to micrometer range. However, as mentioned before, photolithography involves clean room facilities and techniques such as thermal vapor or sputtering material deposition, chemical etching and lift-off steps, or oxygen plasma processes, which assume trained personal and high cost. Most photolithographed microelectrode arrays are made of metal films, although some examples with carbon have also been built using photolithography [6]. Unlike photolithography, screen-printing techniques have an easy fabrication process, but also requires expensive instrumentation. Electrodes are made by transferring suitable inks onto planar substrate materials. This is achieved by forcing the inks through a patterned stencil or mask with a squeegee, followed by appropriate thermal curing. A wide range of inks and substrate materials can thus be used to mass-produce low-cost sensor strips. Commercial carbon and metal (Pt or Au) ink formulations are commonly used for printing the working electrodes, while silver-based inks are used to obtain the reference electrode. All these kinds of ink comprise graphite or metallic particles, a polymer binder, and other additives (for dispersion, printing and adhesion purposes). Each manufacturer has its own formulation; these differences in ink composition and in the printing and curing protocol can greatly affect electron transfer activity and the overall analytical performance of screen-printed sensors. Although screen printing is commonly used for the mass production of electrodes, few papers have reported direct fabrication of miniaturized ISE arrays using this type of screen printed electrodes (SPE) and its application like the photolithographed one [7], in addition limited to a planar configuration. Research into novel, straightforward fabrication methods is crucial for sensor array development. Of particular interest are approaches that do not call for special laboratory equipment or trained personal and that may therefore be produced at low cost.

Here, we describe a reliable, reproducible, low cost and easy method for the simple fabrication of a miniaturized multi-electrode array, which does not require expensive equipment and clean room facilities. The micrometric needle-shape electrodes were integrated in an 8 mm diameter array, designed especially for endoscopic applications, allowing array insertion and monitoring of ischemia in stomach tissue, as was already demonstrated in our previous work [8]. In this paper, a detailed description of the fabrication process of this array is reported. The system is based on a platform comprising 12 metallic pins, which are functionalized as electrodes. After electrode insulation, the top surface was polished to leave delimited the area of the metallic pins electrodes. These were modified with commercial conductive pastes by simple soaking in a thickness controlled film without the use of mask or expensive equipment. The fabricated electrodes were covered with the desired ISE membranes to result in an all-solid state potentiometric sensor array. As a practical example of potentiometric application, we tested the performance of this array for the detection of pH and nitrate. The final purpose of this application is the detection of *Helicobacter pylori* for ulcer monitoring inside the stomach. *Helicobacter pylori* were reported to be present in patients with chronic gastritis and gastric ulcers [9]. The existence of these bacteria causes increase of stomach pH and enhanced concentration of nitrate ions inside stomach [10], which permits its indirect detection with ISEs [11]. However, this work is still in its embryonic stage and preliminary optimization of *in vitro* detection of pH and nitrate are reported in this work.

## 2. Experimental Section

### 2.1. Reagents and Materials

Tetradodecylammonium nitrate (TDAN), hydrogen ionophore IV (octadecyl isonicotinate), poly (vinyl chloride) (PVC) high molecular weight, 2-nitrophenyl octyl ether (NPOE), potassium tetrakis (4-chlorophenyl) borate (KTClPB), potassium ferricyanide (K_3_Fe(CN)_6_) and perfluorinated ion-exchange resin (Nafion) were obtained from Sigma (Madrid, Spain). Tetrahydrofuran (THF), tris(hydroxymethyl)aminomethane (Tris), HCl and KCl were supplied by Panreac (Barcelona, Spain). Carbon and Ag/AgCl ink were purchased from Dupont (Bristol, United Kingdom). MCS 12-series electrode arrays were obtained from Omnetics Connector Corporation (Minneapolis, MN, USA) and epoxy resin (EPOTEK 301-2) was provided by Epoxy Technology (Billerica, MA, USA). ARALDITE 2014-1 was obtained from Farnell (Basel, Switzerland). Polydimethylsiloxane (PDMS) Sylgard 184 Silicone Elastomer preparation kit obtained from Dow Corning (Midland, MI, USA). P4000, P2000, P1000, P400 papers were obtained from Monocomp Instrumentacion (Madrid, Spain).

### 2.2. Instruments and Measurements

Cyclic voltammetry (CV) measurements were done with a CH Instruments electrochemical workstation (CH Instruments, Austin, TX, USA). Potentiometry measurements were performed with a portable PalmSens electrochemical interface (PalmSens, Utrecht, Netherlands). Electrode surface topography was measured using an optical interferometric profiling system (Wyko NT110, Veeco, Plainview, NY, USA) with VSI measurement at 25× magnification. Spin coating of carbon and Ag/AgCl inks was achieved by means of a Laurell Tec Spin Processor WS-400A-6TFM (North Wales, PA, USA). Scanning electron microscope (SEM) images were performed with a Nova NANOSEM 230 FEI microscope (Corvallis, OR, USA) at 5 kV of accelerating voltage with a working distance of 5 mm. SEM images had the magnifications of 3.000× to determine differences in topography between the carbon and Ag/AgCl substrates. pH sensing was done in a solution of Tris adjusted to desired pH by adding controlled concentrations of HCl. Moreover, nitrate sensing was done in a solution of MilliQ water to desired nitrate concentration by adding controlled concentrations of NaNO_3_. The ISE membrane for pH detection was prepared with a mixture of 1 wt% hydrogen ionophore IV, 4 wt% KTClPB, 66.0 wt% 2-nitrophenyloctyl ether, and 29 wt% high molecular weight PVC. A total of 300 mg of these chemicals was dissolved in 3 mL of anhydrous THF [12]. The ISE membrane for nitrate detection was prepared with a mixture of 7.2 wt% nitrate ionophore TDAN, 63.8 wt% NPOE, and 29 wt% PVC. In total 100 mg of these chemicals was dissolved in 2 mL of anhydrous THF [13].

## 3. Results and Discussion

### 3.1. Fabrication of Microelectrodes on an Array

The electrochemical sensor array was composed of 12 electrode pins of beryllium copper alloy. Prior to the modification, they were washed with double deionized (MilliQ) water and dried under nitrogen flow. The sides of the electrode pins were insulated with a commercial resin to reduce the background noise signal and confer mechanical and chemical resistance to the electrodes. A biocompatible resin was chosen for the purpose of implantable sensor application of the array. An easy fabrication method was used for this step, the pins were soaked inside the resin and left to dry in the oven. For that reason, it is necessary a resin with an appropriate density fluid enough to cover each pin separately and dense enough to stay on the pin surface. Different polymeric materials were tried to choose the best candidate. Polydimethylsiloxane (PDMS) is a polymer with appropriate density but, due to its weak mechanical resistance, part of the polymer on the pins was lost after the polishing step. On the other hand, the Araldite 2014-1 polymer showed a high mechanical strength with excellent chemical resistance. However, although the density of the polymer is sufficiently low for a good soaking of the array into the polymer, it is not enough to allow the insulation of pins separately. Finally, the Epoxy 301-2 resin was chosen as the best insulation material, as it overcomes the disadvantages of the other tested polymers. A deeper analysis of the resin choice was given in the following Table 1.

**Figure 1.**
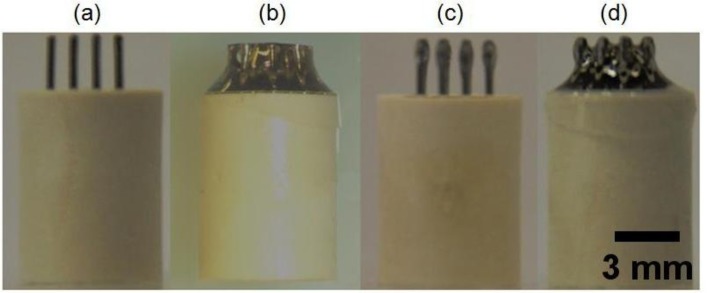
Insulation steps for array fabrication showing the array without insulation (**a**), one-step insulation (**b**), two-step insulation: tip insulation (**c**) and bottom insulation (**d**).

Electrodes were completely covered with the commercial biocompatible EPOTEK 301-2 epoxy resin and the corresponding hardener complex and the mixture was cured at 80 °C for 3 h. The insulation was performed in two steps because one-step deposition of epoxy prevents separation of insulated pins and causes a whole connected array (Figure 1b).

Therefore, first the top parts of the pins were insulated with the epoxy, as shown in Figure 1c. Proper insulation was checked after each step with a multimeter, and the electrodes were soaked in the resin solution until a thick insulation without a conductive signal from the multimeter was achieved. The same steps were then repeated and the bottom part of the pins was covered with the resin so that insulated tips protruded from the resin bulk, as shown in Figure 1d.

**Figure 2.**
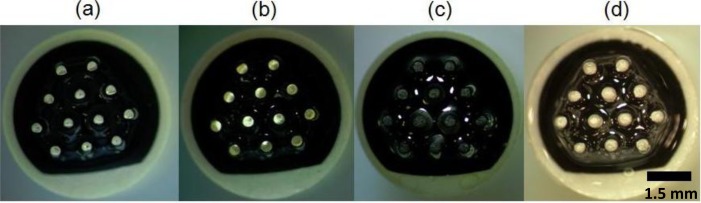
Array before polishing (**a**), after polishing (**b**), after spin coating of carbon ink (**c**) and after spin coating of Ag/AgCl ink (**d**).

This step is crucial to make the platform suitable for the spin-coating process after polishing the tips of the electrodes. The tips of the electrodes were polished with P400, P1000, P2000 and P4000 silicon carbide papers sequentially to remove the insulation layer on the top and to produce a 410-μm diameter flat metal surface (Figure 2b). Afterwards, the tips were cleaned of remaining contaminants by sonication in absolute ethanol for 2 min and dried under nitrogen flow. Afterwards, the pin upper surfaces were protected with a more stable conductive surface with two ink layers of controlled thickness. To deposit these layers in a reproducible and controlled manner, the required inks were spin-coated on a glass slide for a given time and using a determined rpm to achieve the desired thickness. The pins of the array were then soaked in this layer. The full protocol comprised two steps. First, the beryllium copper pins were soaked in carbon paste spin coated under 4000 rpm for 1 min and was left to dry at 130 °C for 6 min (Figure 2c) and then soaked in Ag/AgCl ink in similar manner spin coated at 1000 rpm for 4 min and was left to dry at 130 °C for 6 min (Figure 2d). Intermediate carbon ink layer brings a stable attachment of Ag/AgCl layer on beryllium copper surface.

### 3.2. Characterization of the Fabricated Array

**Figure 3.**
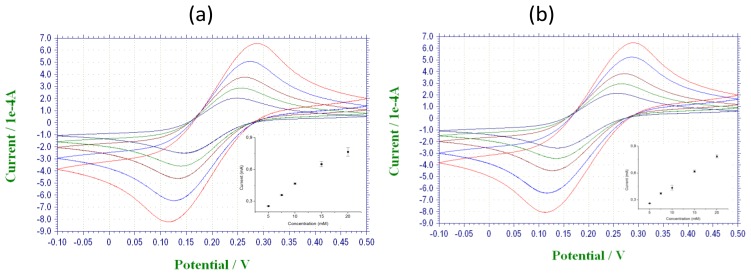
Cyclic voltammetry of carbon (**a**) and Ag/AgCl surfaces (**b**) in ferricyanide solutions (5, 7.5, 10, 15 and 20 mM in 0.1 M KCl).

The electroactive area of carbon and Ag/AgCl surfaces on the fabricated array were characterized by cyclic voltammetry analysis in 0.1 M KCl solution containing a range of concentrations of potassium ferricyanide (Figure 3).

**Figure 4.**
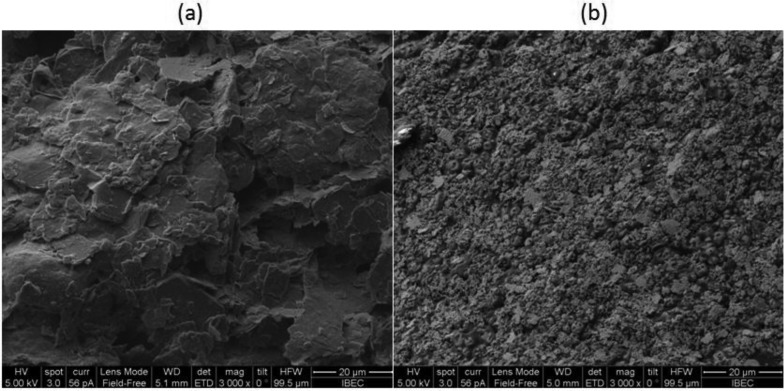
SEM results of carbon (**a**) and Ag/AgCl (**b**) surfaces.

The Randles-Sevčik equation was used to calculate the active surface area of each electrode in the array [14,15]:

(1) $$I_{p}=(2.687\times 10^{5})n^{3/2}\upsilon^{1/2}D^{1/2}AC$$

where I_p_ is the peak current, A is the electroactive area (cm^2^), D is the diffusion coefficient (cm^2^·s^−1^) with the known value of 8.0 × 10^−6^ cm^2^·s^−1^ for ferrocyanide at 25 °C [14,15], C is the concentration of the probe molecule in the bulk solution (mol cm^−3^), υ is the scan rate (0.05 V/s), and n is the number of electrons transferred in the redox event (which in this case is equal to 1). According to the cyclic voltammetry results using the Randles-Sevčik equation, the calculated area for carbon microelectrodes fabricated on an array was 0.182 ± 0.007 cm^2^ and for Ag/AgCl ink deposition with the area of 0.203 ± 0.006 cm^2^ showed good reproducibility with low standard deviation and an efficient method for an easy and low cost fabrication. Considering the diameter of the pins (600 μm) after spin coating, the theoretical area is 0.28 mm^2^, but the surface modification with the ink increases the active surface area 64 times after carbon deposition and 71 times after Ag/AgCl deposition due to the higher roughness of the deposited surface (Figure 4).

**Figure 5.**
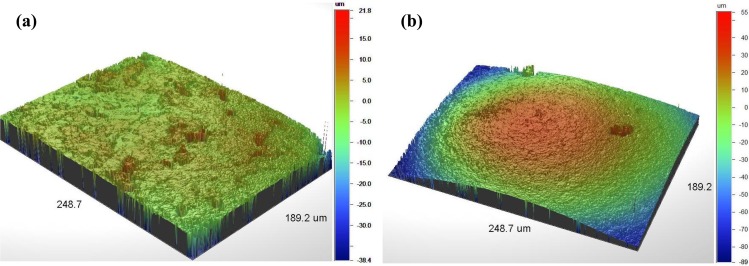
Interferometric profilometry of the (**a**) carbon and (**b**) Ag/AgCl surfaces (at 25× magnification in each case).

The surface topography of carbon and Ag/AgCl layer was examined by optical interferometry. Figure 5 shows 3D topographic maps of each layer, presenting the carbon surface a Root Mean Square (RMS) roughness of 800 ± 200 nm, while the Ag/AgCl surface shown a RMS roughness of 1.2 ± 0.2 μm.

**Figure 6.**
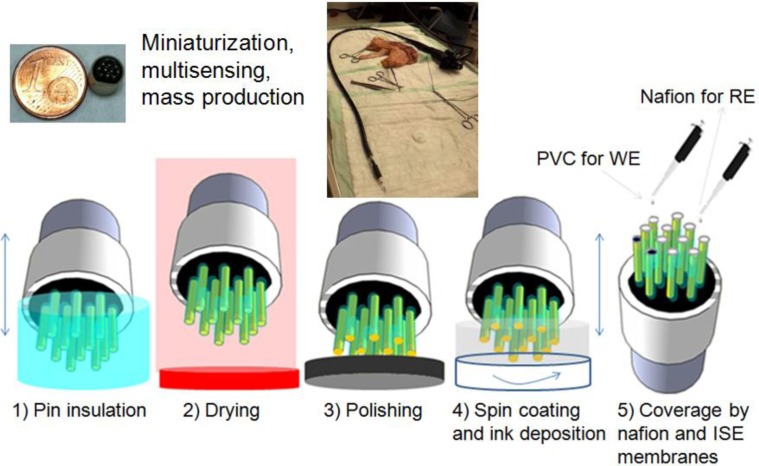
Fabrication steps of all-solid-state pH and nitrate sensors. The inlet picture shows the size of the array and the integration of the sensor array to the commercial endoscope.

Once, the electrodes were fabricated for a stable and efficient way. The ISE sensor was constructed on it. The pin with the Ag/AgCl layer selected to be used as reference electrode (RE) was covered with Nafion to increase the stability of the reference signal and to prevent a reaction between the Ag/AgCl surface and the analyte. After the coverage with Nafion membrane, the electrodes were left for 48 h under vacuum and then dried at 100 °C for 1 h [16]. The electrodes used as working electrodes (WEs) were covered with pH ISE membranes on Ag/AgCl surface or nitrate ISE membranes on carbon surface and left to dry overnight at room temperature for testing the performances of the Ag/AgCl and carbon surfaces as transducer layers. All fabrication steps are summarized in Figure 6. The inlet pictures show the small size of the developed sensor array and the integration of the sensor array to the commercial endoscope.

Standard solutions of pH in 0.1 M Tris buffer solutions and standard solutions of nitrate in MilliQ water were prepared. The sensor arrays were tested at different concentrations of the standard solutions. The potentiometry measurements were performed at low pH (0.7–2.5) for all-solid-state pH sensors [8] and between 10^−5^ to 10^−1^ M nitrate concentrations (NO_3_^−^) for all-solid-state nitrate sensors.

**Figure 7.**
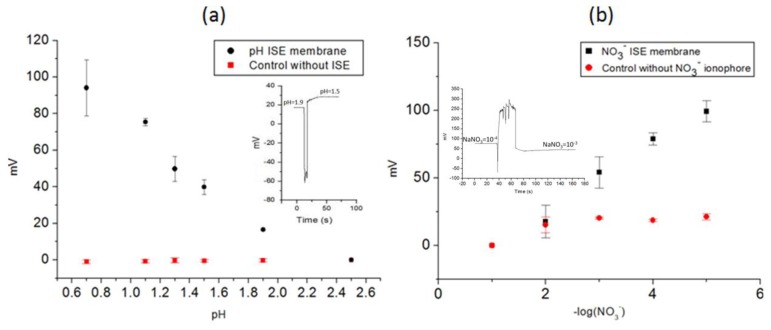
Potentiometry measurements with all-solid-state pH (reproduced with permission of Elsevier from reference [8]) (**a**) and nitrate sensors (**b**) and their control experiments (a, b) were performed to test the effect of the ISE membrane. Inset: A single step change response for the pH and the nitrate ISE.

The potentiometry results in Figure 7 demonstrate the capacity of the sensor array as a nitrate and pH sensors. The control without the ISE membrane showed a flat baseline, thereby proving the selectivity of the ISE membrane for pH sensor (Figure 7a) and the flat beseline for ISE membrane without the nitrate ionophore is proving the importance of ionophore inside the ISE membrane (Figure 7b). Additional studies on the performance of the sensors such as ion interferences and response time analysis have been published in previous paper by the same authors. Additional studies on the performance of the sensors such as ion interferences and response time analysis were also done. As can be appreciated in the kinetic response in the Figure 7 insets, the response time of pH and nitrate sensor are 18 s and 40 s, respectively, fitting to other reported results of all-solid-state pH sensors; between 8 s [17] and 45 s [18]. Spikes of the inset correspond to the change of the vials containing different concentration of ions. The selectivity of the developed pH and nitrate ISE sensor were studied with other cations and anions similar to the analyte of interest; Na^+^, K^+^ for pH ISE sensor and Cl^−^ for nitrate sensors, which are also the cations and anion with higher concentration in the stomach [19]. Selectivity coefficients (K_A,B_) were calculated according to the mixed solution method [20]. Table 2 summarizes the K_A,B_ values for the pH and nitrate ISE sensors.

The longevity of the fabricated ISE sensors is an important parameter for commercialization, since a shorter degradability does not allow a competitive entrance in the market. For this reason, the long term stability of the sensors was tested. For this purpose, a batch of ISE array was tested every week. The arrays kept in open air, were oxidized and degraded after four weeks. On the contrary, the ISE sensor arrays kept under argon were stable up to 12 weeks. The protocol for array fabrication reported in this paper is applicable for the fabrication of arrays with different types of sensors for diverse applications, just by changing the composition of the membranes.

## 4. Conclusions/Outlook

Here, we presented a simple, fast and low cost method to fabricate micro-ISE arrays. The configuration of the fabrication permits easy automation of all the steps for a single adaptation to automatized mass production of sensor fabrication at low cost. The fabricated array has tested with pH and nitrate sensors giving successful results. The array was designed for its integration into commercial endoscopes. The needle shape alignment instead of a planar configuration was used in order to achieve full contact with the tissue. Highly stable electrode surface achieved with the inks permits the efficient detection at low pH and corrosive environment. Modified microelectrodes were characterized by cyclic voltammetry, SEM, and optical interferometry to demonstrate that the fabrication procedure used for the array platform is reproducible. The protocol can be adapted for different applications by tuning ISE membrane composition.

## Figures and Tables

**Table 1.** Different properties of the insulation material were tested.

Polymers	Density	Drying	Hardness	Resistance	Biocompatibility	Remarks
Thermo		80 °C 30 s	Medium	High	High	Bad fit in
Retractile						
Plastics						
PDMS	Low	90 °C 1 h	Low	Medium	High	Too soft
Araldite 2014-1	Medium	60 °C 30 min	High	High	Medium	Good coating
Epoxy 301-2-8OZ	Low	80 °C 3 h	High	High	High	Good coating

**Table 2.** Selectivity coefficients of various interfering ions.

Selectivity coefficients	Na^+^ (NaCl)	K^+^ (KCl)	Cl^−^ (KCl)
K_A,B_	77 × 10^−2^ [8]	78 × 10^−2^ [8]	
K_C,D_			14 × 10^−2^

A = hydrogen cation, B = interfering cation, C = nitrate anion, D = interfering anion.
